# The Quiescin Sulfhydryl Oxidase (hQSOX1b) Tunes the Expression of Resistin-Like Molecule Alpha (RELM-α or mFIZZ1) in a Wheat Germ Cell-Free Extract

**DOI:** 10.1371/journal.pone.0055621

**Published:** 2013-01-31

**Authors:** Wael Gad, Meera G. Nair, Karolien Van Belle, Khadija Wahni, Henri De Greve, Jo A. Van Ginderachter, Guy Vandenbussche, Yaeta Endo, David Artis, Joris Messens

**Affiliations:** 1 Brussels Center for Redox Biology, Brussels, Belgium; 2 Department of Structural Biology, Vlaams Instituut voor Biotechnologie, Brussels, Belgium; 3 Structural Biology Brussels, Vrije Universiteit Brussel, Brussels, Belgium; 4 Lab of Cellular and Molecular Immunology, Vrije Universiteit Brussel, Brussels, Belgium; 5 Myeloid Cell Immunology Lab, Vlaams Instituut voor Biotechnologie, Brussels, Belgium; 6 Cell Free Science and Technology Research Center, Ehime University, Matsuyama, Japan; 7 Department of Microbiology and Institute for Immunology, Perelman School of Medicine, University of Pennsylvania, Philadelphia, Pennsylvania, United States of America; 8 Centre de Biologie Structurale et de Bioinformatique, Structure et Fonction des Membranes Biologiques, Université Libre de Bruxelles, Brussels, Belgium; 9 Department of Pathobiology, School of Veterinary Medicine, University of Pennsylvania, Philadelphia, Pennsylvania, United States of America; 10 Division of Biomedical Sciences, School of Medicine, University of California Riverside, Riverside, California, United States of America; Research Center Borstel, Germany

## Abstract

**Background:**

Although disulfide bond formation in proteins is one of the most common types of post-translational modifications, the production of recombinant disulfide-rich proteins remains a challenge. The most popular host for recombinant protein production is *Escherichia coli*, but disulfide-rich proteins are here often misfolded, degraded, or found in inclusion bodies.

**Methodology/Principal findings:**

We optimize an *in vitro* wheat germ translation system for the expression of an immunological important eukaryotic protein that has to form five disulfide bonds, resistin-like alpha (mFIZZ1). Expression in combination with human quiescin sulfhydryl oxidase (hQSOX1b), the disulfide bond–forming enzyme of the endoplasmic reticulum, results in soluble, intramolecular disulfide bonded, monomeric, and biological active protein. The mFIZZ1 protein clearly suppresses the production of the cytokines IL-5 and IL-13 in mouse splenocytes cultured under Th2 permissive conditions.

**Conclusion/Significance:**

The quiescin sulfhydryl oxidase hQSOX1b seems to function as a chaperone and oxidase during the oxidative folding. This example for mFIZZ1 should encourage the design of an appropriate thiol/disulfide oxidoreductase-tuned cell free expression system for other challenging disulfide rich proteins.

## Introduction

**Figure 1:**
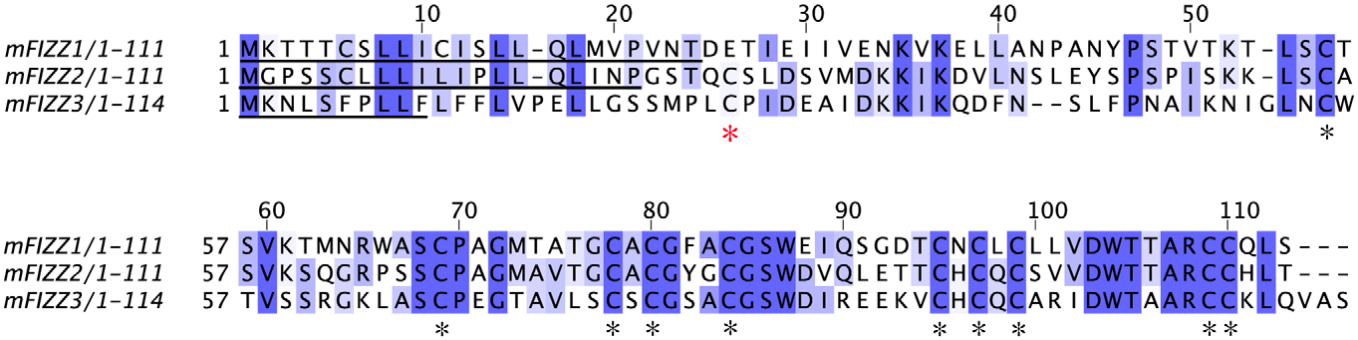
The cysteines in the resistin family are highly conserved. A ClustalW alignment [50] of the mFIZZ protein amino acid sequences is shown. Gene Bank accession numbers are for mFIZZ1, AF205951; mFIZZ2, Q99P86 and mFIZZ3 Q99P87. The conserved residues have been coloured in blue grade (conservation level 30%). The conserved cysteines are marked with black asterisks, and the signal peptides are underlined. The position of the extra N-terminal cysteine in mFIZZ2 and mFIZZ3 is indicated with a red asterisk. The extra N-terminal cysteine is thought to be involved in an inter-molecular disulfide bond in mFIZZ2 [27].

Resistin is part of the FIZZ (found in inflammatory zones) family of genes, and was first characterized in murine models where it has been extensively studied as a potential link between type II diabetes and obesity [1]. The murine FIZZ gene family consists of three related gene products: i) in steady state, mFIZZ1 or RELM-α is found in adipocytes and lung tissue, ii) mFIZZ2 or RELM-β found in the gastrointestinal tract, and iii) mFIZZ3 or resistin found in adipocytes (Figure 1). These are small (∼12 kDa) secreted proteins with a conserved cysteine pattern [2], [3]. mFIZZ1 shows 41% amino sequence identity with resistin (mFIZZ3) and 51% with RELM-β (mFIZZ2), which has an unusual multimeric structure [4]. In this study, we will use the mouse protein mFIZZ1, which has an established role in influencing innate and adaptive immune responses as a negative regulator of type 2 inflammation [3], [5]. The structure of mFIZZ1 is not known and it is a 10-cysteine containing protein that has to form five disulfide bonds. Disulfide bonds are here critical for proper protein folding, stability and activity, and it is known that about 15% of all the human proteins are predicted to form disulfide bonds [6], [7]. They are formed by the oxidation of sulfhydryl groups between two cysteines resulting in a covalent bond after the translocation of the native polypeptide chains to the extra cytoplasmic compartments of the cell [6]. Despite many years of research, the mechanistic features and driving forces of several oxidative protein folding systems are still not fully understood and are a matter of debate. Complex enzymatic systems control the oxidation state of cysteine residues in proteins, either by reducing or oxidizing depending on the identity of the protein target, the subcellular compartment, and the redox properties of the environment. In the periplasm of *E. coli*, the Dsb (disulfide bond)-family (DsbA, DsbB, DsbC, DsbG and DsbD) of proteins are involved [8]. In the eukaryotic mitochondrial innermembrane space and in the endoplasmic reticulum (ER), similar disulfide relay mechanisms with distinct mechanistic properties introduce disulfide bonds in polypeptide chains [9]. In the endoplasmic reticulum, protein disulfide isomerase (PDI) has, in addition to an isomerase and a reductase function, an oxidase function [10]. PDI is recycled via Ero1p, a FAD-containing oxidase that uses oxygen as final electron acceptor [11]. In yeast, an alternative Ero1-independent disulfide bond formation pathway uses Erv2p, a FAD-binding sulfhydryl oxidase that introduces disulfide bonds [12]. In the endoplasmic reticulum of non-fungal eukaryotes, proteins with homologous Erv2p-domains are known as QSOX (Quiescin–Sulfhydryl Oxidase) [13], for which two variants (hQSOX1 and hQSOX2) are described in humans [14]–[16]. Two splice variants of the human hQSOX1 gene have been reported, one which encodes for the 747 amino acids hQSOX1a, and another shorter one that lacks the transmembrane helix, which encodes for the 604 amino acids hQSOX1b [13]. These sulfhydryl oxidase proteins consist of a fusion of two functional domains [17]. At the N-terminus, QSOX contains a dithiol/disulfide oxidoreductase domain [18] related to PDI. Towards its C-terminus, QSOX has a sulfhydryl oxidase domain [19], which forms disulfides *de novo* [20]. These sulfhydryl oxidases catalyze disulfide bond formation by reduction of molecular oxygen to hydrogen peroxide.

We have chosen for a fast and easy to tune expression system, the eukaryotic cell-free translation system based on the wheat germ embryo (WGE) [21]. Except for mRNA, all the components for translation are here stored in a dried state, ready for protein synthesis as soon as germination starts [22]. We challenged this expression system with a 10-cysteine containing protein, mFIZZ1 that has to form five disulfide bonds, and with mFIZZ1′, which is the mFIZZ1 protein with its 2.5 kDa signal peptide that contains an extra 2 cysteines. We investigated the role of hPDI and hQSOX1b as a possible protein folding catalyst for the expression these proteins, and showed for the first time expression of soluble and active monomeric mFIZZ1 using co-expression with hQSOX1b.

## Results

### mFIZZ1 expressed in *E. coli* is always found in inclusion bodies

**Figure 2:**
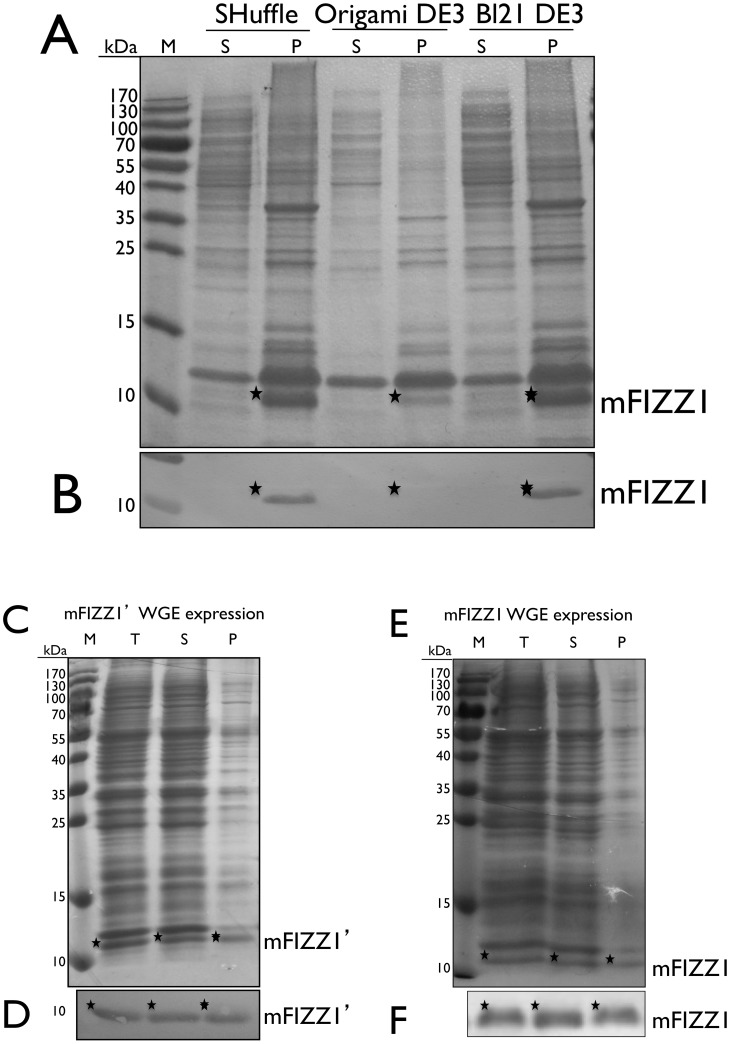
mFIZZ1 soluble expressed using wheat germ extract. (**A**) The expression of mFIZZ1 in SHuffle™ T7, Origami DE3 and BL21 DE3 analysed on non-reducing 15% SDS-PAGE stained with Coomassie Brilliant Blue. (**B**) Strip of the immunoblot from the SDS-PAGE in (**A**) developed with anti-His antibody is shown. (**C**) The expression of mFIZZ1′ with wheat germ extract analysed on non-reducing 15% SDS-PAGE stained with Coomassie Brilliant Blue. (**D**) Strip of the respective immunoblot of (**C**) developed with anti-His antibody is shown. (**E**) The expression of mFIZZ1 with wheat germ extract analysed on non-reducing 15% SDS-PAGE stained with Coomassie Brilliant Blue. (**F**) Strip of the respective immunoblot of (**E**) developed with anti-His antibody is shown. As marker (M) the PageRuler™ pre-stained Protein Ladder (Fermentas) is used. P = pellet, S = soluble fraction and T = total. The corresponding bands are indicated with an asterisk.

We first decided to target the expression of mFIZZ1 with an N-terminal His-tag to the cytoplasm of *E. coli* SHuffle™ T7 Express, Origami™ DE3 and BL21 DE3. Soluble and insoluble fractions were evaluated on non-reducing 15% SDS-PAGE (Figure 2A) and on immunoblot using anti-His antibody (Figure 2B). On a non-reducing SDS-PAGE the band of mFIZZ1+ His-tag migrates at 11 kDa, consistent with its calculated mass. Soluble expression of mFIZZ1 expression in *E. coli* was not successful. Only in the insoluble pellet fraction a clear band of mFIZZ1 was detected. Decreasing the temperature of expression did not help to produce soluble protein, and periplasmic expression in *E. coli* of mFIZZ1 always resulted in inclusion bodies (data not shown). The Dsb disulfide bond formation machinery of *E. coli* seems to be unable to cope with this multiple cysteine containing polypeptide chain. Also the expression of recombinant resistin (mFIZZ3) using a pQE-31 vector resulted in the expression in inclusion bodies in *E. coli* JM109 [23].

### A wheat germ in vitro translation system expresses mFIZZ1 in the soluble fraction

We decided to use a fast and easy to tune expression system, the eukaryotic cell-free translation system based on the wheat germ embryo [21]. In the embryos, all the components for translation, except for mRNA, are stored in a dried state, ready for protein synthesis as soon as germination starts [22]. This system is expected to have the ability to synthesize eukaryotic multi-domain proteins in a folded state [22], and we challenged this expression system with mFIZZ1 and mFIZZ1′. Important to note is that the signal peptide contains two extra cysteine residues. Gene fragments encoding for mFIZZ1 and mFIZZ1′ were cloned into pEU-His vector. The plasmid DNA (2 µg) was transcribed for 6 h at 37°C using SP6 RNA polymerase, the mRNA was cooled down and checked on agarose gel. For translation, the mRNA (10 µl) was mixed with wheat germ extract (10 µl) and added carefully to form the bottom layer. The amino acid mixture (206 µl) was added to form the upper layer. The total reaction mixture (226 µl) was translated for 20 h at 15°C in a 96-well plate. The expression of mFIZZ1 and mFIZZ1′ were evaluated on non-reducing 15% SDS-PAGE (Figure 2C and 2E) and immunoblot with anti-His antibody (Figure 2D and 2F). The indicated band was excised from the gel and mass spectrometric analysis of the peptides after a tryptic digest identified the band as mFIZZ1. For the first time, we were able to express mFIZZ1 with and without signal peptide in the soluble fraction. This was not completely surprising, as previous studies have shown that an eukaryotic expression system is more suited for the expression of recombinant eukaryotic proteins [22]. However, still part of the mFIZZ1 proteins were not correctly folded and found in the insoluble pellet.

### The sulfhydhryl oxidase hQSOX1b folds mFIZZ1 and mFIZZ1′

**Figure 3:**
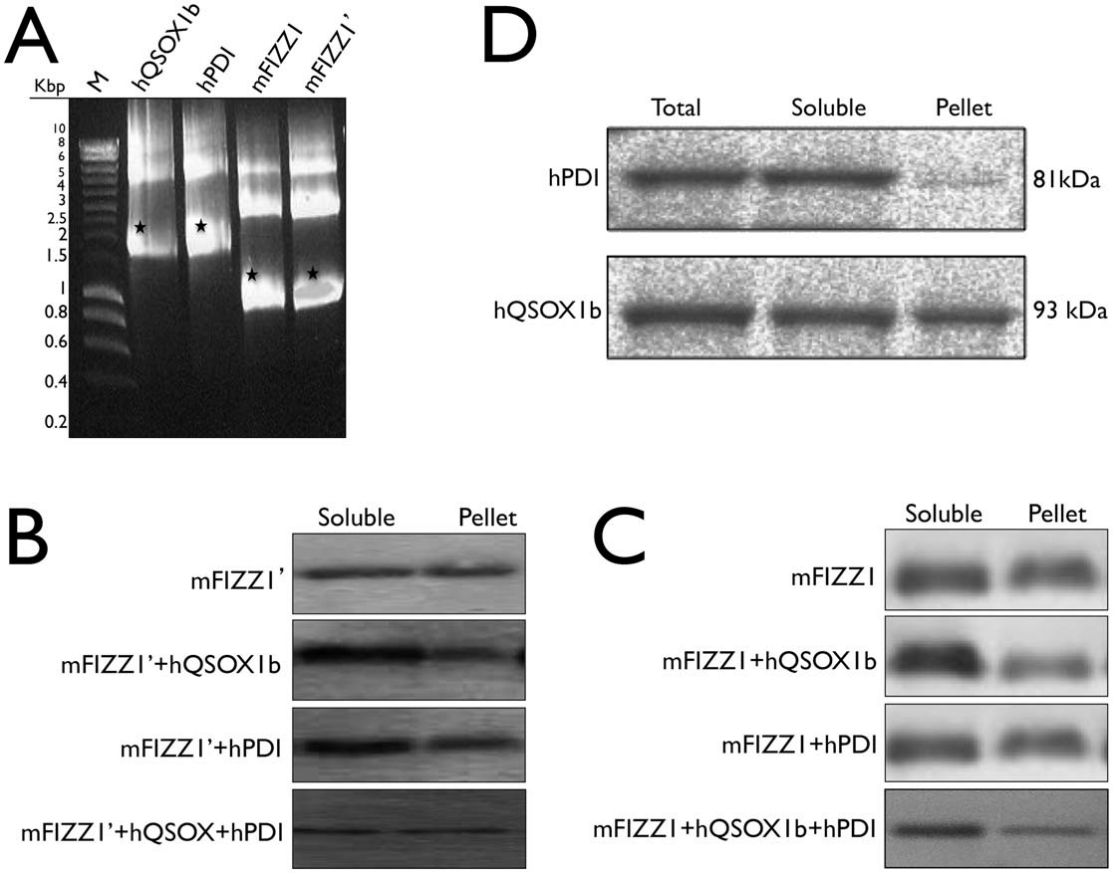
Co-expression with hQSOX1b increased the soluble expression of mFIZZ1. (**A**) Ethidium bromide stained agarose gel with the mRNA of hQSOX1b, hPDI, mFIZZ1 and mFIZZ1′ after transcription is shown. (**B**) An immunoblot developed with anti-His antibody shows the expression of mFIZZ1′ without thiol-disulfide oxidoreductase, with hQSOX1b, with hPDI and with hQSOX1b + hPDI. (**C**) An immunoblot developed with anti-His antibody shows the expression of mFIZZ1 without thiol-disulfide oxidoreductase, with hQSOX1b, with hPDI and with hQSOX1b + hPDI. (**D**) An immunoblot developed with anti-GST antibody shows the expression of both hQSOX1b+GST (93 kDa) and hPDI+GST (81 kDa) after the co-expression reaction with mFIZZ1.

In our attempt to increase the amount of soluble mFIZZ1, we evaluated the co-expression of mFIZZ1 and mFIZZ1′ with the helper proteins hQSOX1b and hPDI, both eukaryotic thiol/disulfide oxidoreductases. We cloned hQSOX1b and hPDI into a pEU-GST vector and evaluated the co-expression of hQSOX1b, hPDI and both thiol/disulfide oxidoreductases together with mFIZZ1 or mFIZZ1′ under the same experimental cell free expression conditions. The plasmid DNA of the four constructs (2 µg) was transcribed for 6 h at 37°C using SP6 RNA polymerase, the mRNA of the plasmids were cooled down and checked on agarose gel (Figure 3A). In all experiments, the expression levels of hQSOX1b and hPDI during the reactions were checked by immunoblot using anti-GST antibody (Figure 3D). The isomerase hPDI was soluble expressed; and for hQSOX1b, more than 50% of the expressed protein was in the soluble fraction.

Interestingly, when we compared the expression of mFIZZ1 and mFIZZ1′ by immunoblot (Figures 3B and 3C), we observed an increase of mFIZZ1 (70%) and mFIZZ1′ (65%) in the soluble fraction when we co-expressed in the presence of the oxidase hQSOX1b (Table 1). Co-expression with hPDI also increased the soluble expression (51% and 59%), but not as much as compared to co-expression with hQSOX1b. On the other hand, combining hPDI and hQSOX1b does not increase soluble fraction of mFIZZ1 and mFIZZ1′ (Table 1). Combining the plasmids results in low expression due to the competition during translation of the three plasmids for the same amount of components in the reaction mixture.

**Table 1:** Scanned protein bands (%) of the immunoblots of the figures 2B and 2C.

protein band on immunoblot	soluble (%)	pellet (%)
mFIZZ1	44	56
mFIZZ1 + hQSOX1b	70	30
mFIZZ1 + hPDI	51	49
mFIZZ1 + hQSOX1b + hPDI	58	42
mFIZZ1′	55	45
mFIZZ1′ + hQSOX1b	65	35
mFIZZ1′ + hPDI	59	41
mFIZZ1′ + hQSOX1b + hPDI	50	50

### mFIZZ1 and mFIZZ1′ are monomeric proteins with all disulfide bonds formed

In the next step, we purified mFIZZ1 and mFIZZ1′ in the presence and absence of hQSOX1b using Ni^2+^ Immobilized Metal Affinity Chromatography (IMAC). We obtained a final yield of ∼300 µg mFIZZ1′ in a 6 ml wheat germ reaction. Co-expression with hQSOX1b resulted in a final yield of ∼120 µg for a 6 ml reaction mixture, which might be due to the translation from two plasmids with the same and limited amount of compounds. For mFIZZ1 the yield was ∼340 µg, while in the presence of hQSOX1b it was ∼160 µg.

**Figure 4:**
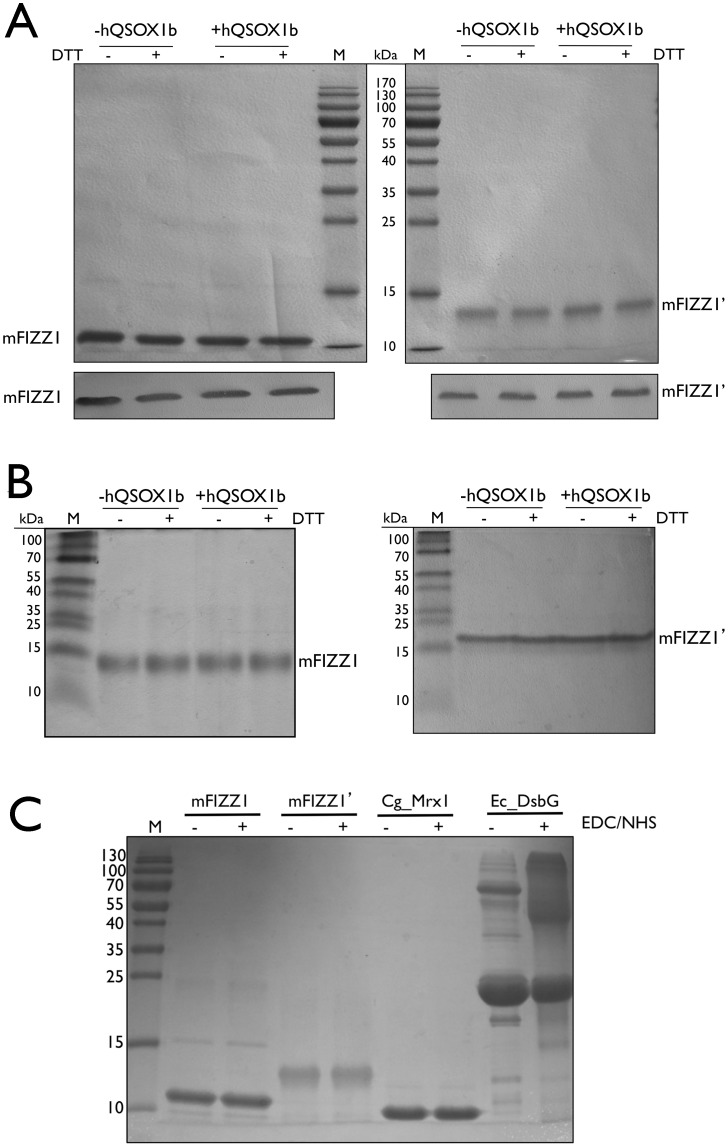
mFIZZ1 and mFIZZ1′ are highly pure and monomeric. (**A**) CBB-stained 15% SDS-PAGE of the purified mFIZZ1′ and mFIZZ1 co-expressed with and without hQSOX1b under reducing and non-reducing conditions followed by immunoblot developed with anti-His antibody. The samples are highly pure and proteins migrate at the same position under reducing and non-reducing conditions, indicating that no intermolecular disulfide bonds are formed. (**B**) Basic native PAGE of the purified mFIZZ1 and mFIZZ1′ co-expressed with and without hQSOX1b under reducing and non-reducing conditions. The protein bands for mFIZZ1 (pI 4.81) and mFIZZ1′ (pI 5.18) migrate on a slightly different position due to their pI. No major multimeric bands are observed. (**C**) A CBB-stained 15% non-reducing SDS-PAGE of mFIZZ1 and mFIZZ1′ co-expressed with hQSOX1b after cross-linking with a mixture EDC/NHS for 3 h at room temperature is shown. As negative and positive controls, monomeric *Corynebacterium glutamicum* mycoredoxin-1 (Cg_Mrx1) and dimeric *Escherichia coli* DsbG (Ec_DsbG) were used. Monomeric Cg_Mrx1 migrates after crosslinking as monomer. For dimeric Ec_DsbG (present as a mixture between monomer and dimer on SDS-PAGE), bands shift from the monomeric and the dimeric migration position to a higher molecular weight positions. For both mFIZZ1 and mFIZZ1′ no shift to a higher molecular weight is observed. As marker (M) in panels **A**, **B** and **C**, the PageRuler™ pre-stained Protein Ladder (Fermentas) is used, which contains SDS.

**Figure 5:**
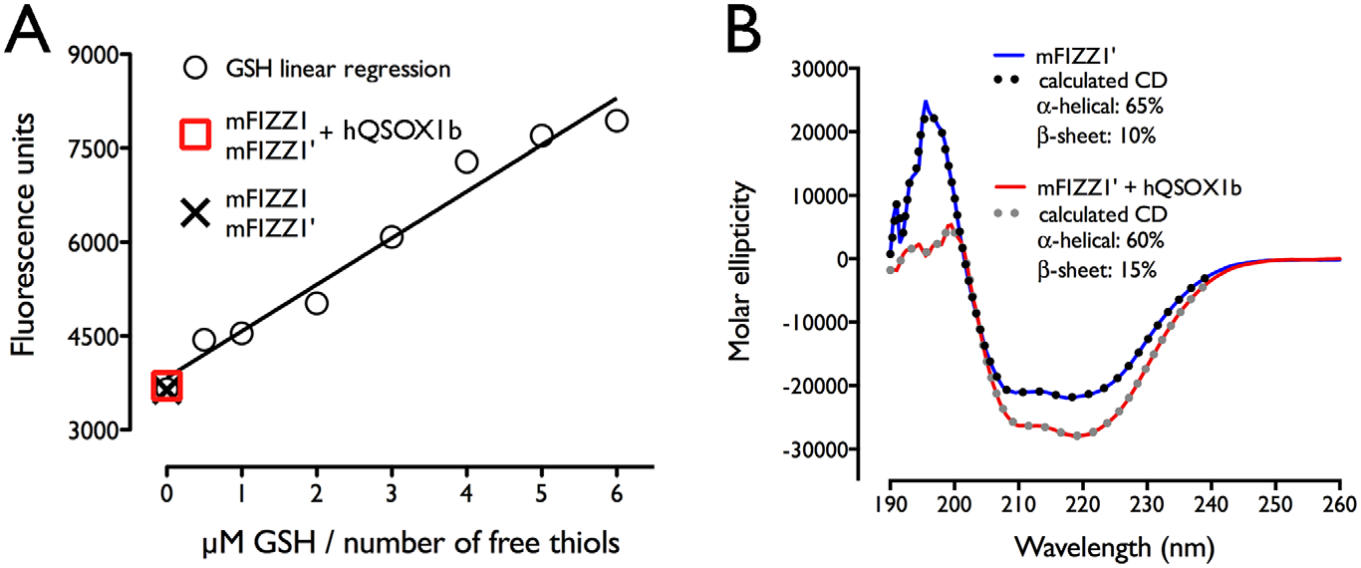
mFIZZ1′ has a high helical content and all the intramolecular disulfide bonds are formed. (**A**) The results of a Thiostar assay using glutathione as a standard are shown. mFIZZ1′ and mFIZZ1 prepared with and without hQSOX1b show no free thiols. (**B**) The CD-spectrum of mFIZZ1′ produced with and without hQSOX1b showed a double minimum at 208 and 222 nm characteristic for α-helical proteins.

Purified proteins were evaluated on 15% SDS-PAGE under reducing and non-reducing conditions followed by immunoblot using an anti-His antibody (Figure 4A). The samples are highly pure and proteins migrate at the same position under reducing and non-reducing conditions, indicating that no intermolecular disulfide bonds are formed. This is different compared to what has been reported for mFIZZ3, which forms a disulfide-linked homodimer [24]. We confirmed this observation by checking the number of free thiols in a Thiostar assay. With an increasing amount of glutathione as a standard, we showed that both mFIZZ1 and mFIZZ1′ prepared with and without hQSOX1b showed no free thiols (Figure 5A).

As mFIZZ1 and mFIZZ1′ might still form non-disulfide linked multimers in solution, we also analyzed the proteins on native gels (Figure 4B) under reducing and non-reducing conditions. Non-boiled samples of mFIZZ1 (pI 4.81) and mFIZZ1′ (pI 5.18) migrate based on their intrinsic charge at pH 8.9 on slightly different positions as a monomer and no multimeric bands were observed. Moreover, we performed a crosslinking experiment with mFIZZ1 and mFIZZ1′ produced in the presence of hQSOX1b. If mFIZZ1 or mFIZZ1′ were multimers in solution, we expect to observe a band shift in the presence of cross-linker on SDS-PAGE. We incubated samples of mFIZZ1 and mFIZZ1′ for 3 hours with EDC (1-ethyl-3-[3-dimethylaminopropyl]carbodiimide hydrochloride) to crosslink carboxylates (-COOH) to primary amines (-NH2) in the presence of N-hydroxysuccinimide (NHS) to stabilize the amine-reactive intermediate [25], [26]. Boiled samples incubated without and with EDC/NHS were evaluated on SDS-PAGE next to positive and negative control proteins for which the oligomeric state is known (Figure 4C). Both mFIZZ1 and mFIZZ1′ migrate as a single band and no band shifts were observed like for DsbG from *Escherichia coli*. Our results strongly indicate that mFIZZ1 and mFIZZ1′ are monomeric in solution. For the experiment in the absence of hQSOX1b similar results were obtained. In mFIZZ2 and mFIZZ3, an extra N-terminal cysteine is present (Figure 1), which in the structure of the related human FIZZ2 [4] is involved in intermolecular disulfide bond formation. In mFIZZ1, this N-terminal cysteine is not present, which might explain why recombinant mFIZZ1 and mFIZZ1′ are monomeric proteins with no intermolecular disulfide bonds. Our result confirms the observation of Banerjee *et al.* [27]. They showed disulfide-linked dimerization for FIZZ2 and FIZZ3 via the N-terminal cysteine, and characterized FIZZ1 as a monomer.

### Folding in the presence of hQSOX1b decreases the alpha-helical content of mFIZZ1′

We checked secondary structure of recombinant purified mFIZZ1′ in the presence and absence of hQSOX1b with far-UV circular dichroism (CD). The CD-spectra of mFIZZ1′ produced in the presence and absence of hQSOX1b showed a double minimum at 208 and 222 nm characteristic for α-helical proteins, indicating that the protein produced contains a significant amount of secondary structure (Figure 5B). We used the CDSSTR algorithm [28] from DiChroWeb (http://dichroweb.cryst.bbk.ac.uk) [29] to determine the secondary structure. Both calculated CD curves (mFIZZ1′ and mFIZZ1′+hQSOX1b) gave an almost perfect fit with nrsmd values of 0.004 and 0.001, respectively. For mFIZZ1′ produced in the presence of hQSOX1b, the best fit resulted in an α-helical content of 60% and a β sheet content 15%, while in the absence of hQSOX1b an α-helical content of 65% and β sheet content of 10% were obtained. Compared to resistin (mFIZZ3) [23] and RELM-β (human FIZZ2) [4], the α-helical content of mFIZZ1′ is much higher. mFIZZ3 contains 36% α-helical content and 9% β-sheet [23], whereas human FIZZ2 has a multimeric structure with carboxy-terminal disulfide-rich β-sandwich “head” domain (38%) and an amino-terminal α-helical “tail” segment (12%) (PDB code 1HR7) [4]. Although resistin proteins have a clearly conserved cysteine pattern (Figure 1), they have clearly different structural folds and mFIZZ1′ seems to be predominantly helical. Intriguing, the quiescin sulfhydryl oxidase hQSOX1b has an impact on the folding of mFIZZ1′ decreasing its helical content by 5%.

### Only hQSOX1b co-expressed mFIZZ and mFIZZ1′ are biologically active

**Figure 6:**
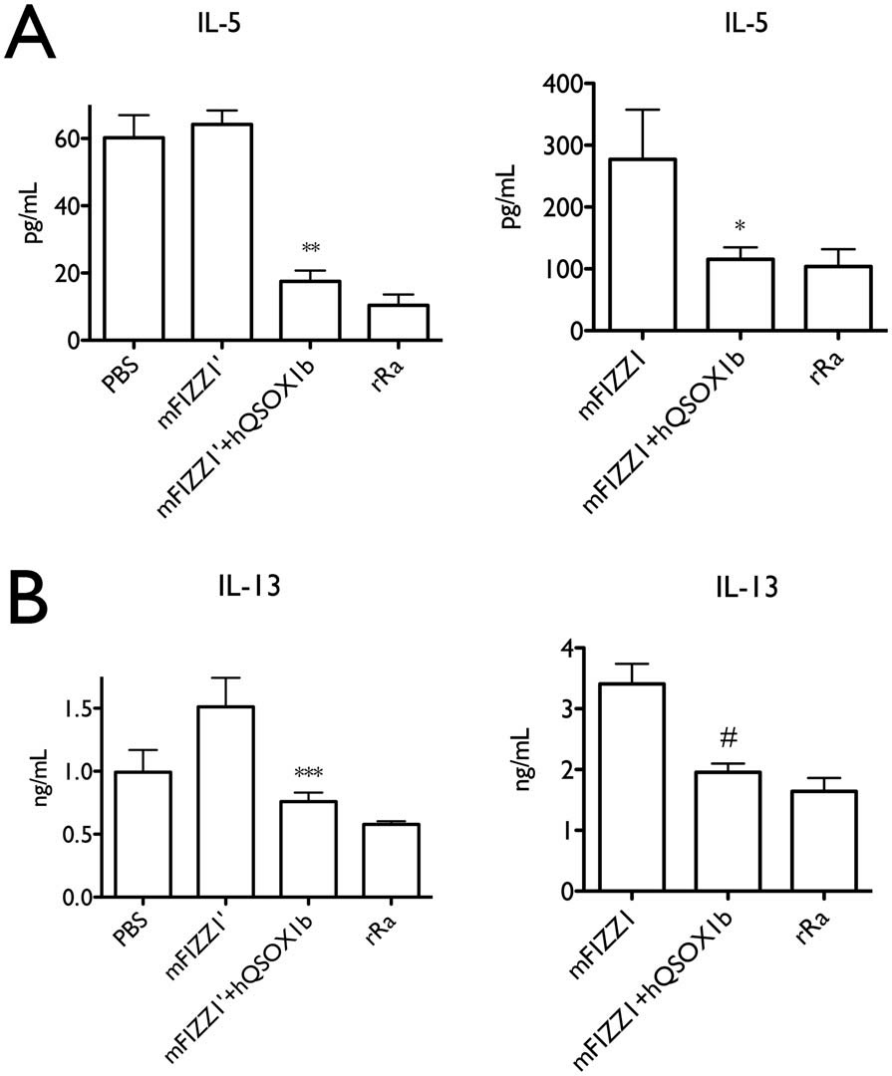
mFIZZ1′ and mFIZZ1 decreased the IL-13 and IL-5 secretion of splenocytes. Splenocytes were cultured at 200,000 cells/well and activated under Th2 permissive conditions for 4 days. Recombinant mFIZZ1′ and mFIZZ1 expressed with or without hQSOX1b were used at 200 ng/ml. rRa is the bacterial recombinant FIZZ1 (200 ng/ml) from (Peprotech) and PBS is the control. (**A**) Recombinant mFIZZ1′ and mFIZZ1 co-expressed with hQSOX1b significantly decreased the IL-5 secretion compared to the proteins expressed alone. (**B**) Recombinant mFIZZ1′ and mFIZZ1 co-expressed with hQSOX1b decreased the IL-13 secretion compared to the proteins expressed alone. ***P<0.001; **P<0.01; *P<0.05; #P = 0.07. Results are representative of three independent experiments for mFIZZ1′ and two independent experiments for mFIZZ1, and statistical analysis was performed by two-way ANOVA.

We next evaluated the ability of purified mFIZZ1′ and mFIZZ1 to suppress Th2 cytokine expression by splenocytes [30]. Recombinant proteins expressed with hQSOX1b or without hQSOX1b were used at a concentration of 200 ng/ml. As negative and positive controls, we employed PBS-treated cells and the commercially available bacterial derived recombinant mFIZZ1 (rRa) from Peprotech at the same concentration [30]. Although the samples of mFIZZ1′ prepared in the absence of the quiescin sulfhydryl oxidase hQSOX1b showed secondary structure with no free thiols (Figures 5 A and B), no significant activity was measured compared to PBS-control (Figure 6). This might indicate that the disulfides in this preparation were not correctly formed or that other post-translational modifications like overoxidation to sulfenic, sulfinic or sulfonic occurs in the absence of hQSOX1b.

In contrast, recombinant mFIZZ1 and mFIZZ1′ co-expressed with hQSOX1b significantly decreased IL-5 and IL-13 secretion, and the same values for the bacterially derived protein (rRA) were obtained (Figure 6). The concentration of mFIZZ1′ and mFIZZ1 used is reflective for the levels observed *in vivo* [31], highlighting the physiological relevance of employing biologically active mFIZZ1 and mFIZZ1′ that was made when co-expressed with hQSOX1b. Together these data demonstrate that for mFIZZ1 and mFIZZ1′ activity on splenocytes, all disulfide bonds need to be correctly connected and that the sulfhydryl oxidase hQSOX1b plays an essential role in the oxidative folding process.

### hQSOX1b has oxidase and chaperone activity

From the previous results, it is not clear whether hQSOX1b works as an oxidase, an isomerase, or a chaperone. We used an *E. coli* RNase I activity assay [32] to figure out the specific function of hQSOX1b by using a disulfide number molar excess of recombinant hQSOX1b in comparison with DsbA, DsbC and hPDI. We showed in the past successful *in vitro* folding of RNase I with DsbA and DsbC under these condition [32], as both Dsb enzymes are not regenerated after a single catalytic event [33].

**Figure 7:**
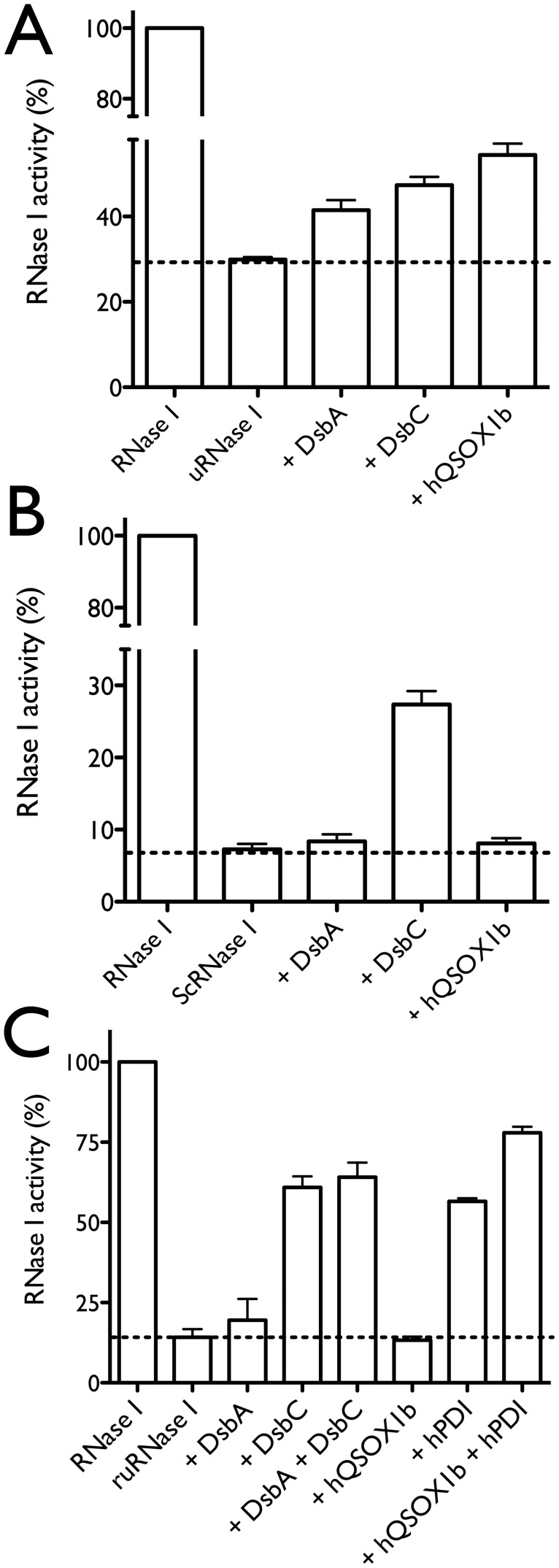
hQSOX1b has chaperone activity and cooperates with PDI to fold reduced unfolded RNase I. The mean values and the standard deviation of the RNase I activity of three independent experiments are shown. (**A**) Chaperone assay with unfolded RNase I (uRNase I). hQSOX1b helps to fold unfolded RNase I (**B**) Isomerase assay with scrambled RNase I (scRNase I). hQSOX1b did not show isomerase activity, while the isomerase DsbC partially rescues the RNase I activity. (**C**) Oxidase assay with reduced unfolded RNase I (ruRNase I). Combining hQSOX1b with hPDI, and DsbA with DsbC results in the highest oxidative folding efficiency. hQSOX1b on its own does not catalyse the folding of reduced unfolded RNase I. In each assay, the background value is indicated with a dotted line.

In the chaperone activity assay (Figure 7A), unfolded RNase I was pre-incubated with hQSOX1b for 3 min at 15°C in a final concentration of 0.5 µM unfolded RNase I before measuring RNase activity. The quiescin sulfhydryl oxidase hQSOX1b showed the highest chaperone activity compared to DsbA and DsbC of *E. coli.* (Figure 7A). Decreasing the concentrations of hQSOX1b from 5 µM to 50 nM gives similar RNase I activity up to 0.5 µM (Table 2). A further decrease of the hQSOX1b concentration below the concentration of RNase I results in background RNase I activity. At least the same concentrations of hQSOX1b as RNase I are needed to refold RNase I, which indicates chaperone activity for hQSOX1b.

**Table 2:** The concentration variation of hQSOX1b in the chaperone-folding assay.

RNase I	uRNase I*	hQSOX1b	RNase I	Relative activity
(µM)	(µM)	(µM)	A659 nm/min	(%)
0.5	-	-	0.352	100.0
-	0.5	-	0.051	30.9
-	0.5	5	0.2164	61.5
-	0.5	1	0.2126	54.9
-	0.5	0.5	0.1955	55.5
-	0.5	0.05	0.0508	16.5

* uRNase I = unfolded RNase I.

We next tested the isomerase activity of hQSOX1b on scrambled RNase I of *E. coli* with its disulfide bonds wrongly connected (0.5 µM scRNase I) (Figure 7B). hQSOX1b did not show isomerase activity, while DsbC was able to partially refold RNase I [32].

Finally, we tested the oxidase activity with reduced and unfolded RNase I (1 µM ruRNase I) incubated for 3 min at 25°C. The combination of the folding catalysts hQSOX1b + hPDI and DsbA + DsbC results in the highest oxidative folding efficiency, with the largest contribution for the isomerases hPDI and DsbC (Figure 7C). The *in vitro* oxidase activity of DsbC [32], [34] and hPDI have already been shown [35]. Important to note is that for the refolding of ruRNase I only catalytic concentrations of 50 nM hQSOX1b were used. The electrons are directly passed via its FAD-group to O_2_ to form H_2_O_2_, and hQSOX1b does not need to be recycled by a downstream pathway like for the Dsb-proteins. As 50 nM of hQSOX1b has no chaperone effect on unfolded RNase I (Table 2), hQSOX1b should function as an oxidase in this experiment. On the other hand, 50 nM or 5 µM hQSOX1b alone do not catalyze the folding of the ruRNase I (Figure 7C). These results suggest that hQSOX1b might cooperate with hPDI present in the wheat germ extract [36] to correctly fold mFIZZ1. Also, Rancy and Thorpe showed that QSOX and PDI work together to generate native pairings in two unfolded reduced proteins [37].

## Discussion

All together, well-folded, soluble full-length monomeric mFIZZ1 is produced in a wheat germ cell-free expression system. The protein mFIZZ1 forms five disulfides and the protein mFIZZ1′ with its signal peptide forms six disulfides. Both mFIZZ1 proteins are only biologically active in the presence of hQSOX1b. Indeed, in the absence of hQSOX1b, all disulfide bonds are formed, but no bioactivity was detected. We might conclude that these non-active forms are disulfide-scrambled with a higher helical content (CD-spectrum) and a different structural fold. The fact that mFIZZ1′ with its predicted signal peptide gave a similar result came as a surprise. *In vivo*, the signal peptide of mFIZZ1 is removed by signal peptidase enzymes [38].

The quiescin sulfhydryl oxidase hQSOX1b seems to have chaperone and oxidase activity.

The chaperone activity of hQSOX1b might prevent incorrect disulfide formation or might even prevent post-translational over-oxidation. As an oxidase, hQSOX1b needs to work in concert with the isomerase hPDI. To our knowledge, this is the first time that chaperone activity has been described for hQSOX1b. Perhaps not a complete surprise as this quiescin sulfhydryl oxidase consists N-terminal Trx-domains like in PDI. For PDI, molecular chaperone activity has been reported for the folding of procollagen [39] and for glyceraldehyde-3-phosphate dehydrogenase [40]. More examples with several disulfide bond free substrate proteins are needed to further confirm the chaperone activity of hQSOX1b. Moreover, it has recently been shown that the chaperone activity of PDI and the overall conformation of human PDI are redox-regulated [41]. As such, in the experiment where we combined hQSOX1b with hPDI, the produced H_2_O_2_ from the formation of disulfide bonds by hQSOX1b may have increased the chaperone activity of hPDI. In this case hPDI might correct disulfide bonds as an isomerase and its chaperone activity will increase the moment more disulfide bonds are introduced by the oxidase hQSOX1b, which would result in a clear working together of those two thiol/disulfide oxidoreductase. At this stage, however, it is not clear which of the properties of hQSOX1b and hPDI are needed to correctly fold mFIZZ1 and mFIZZ1′ into a biological active protein. *In vivo*, the functional role of hQSOX1b in the folding of mFIZZ1 protein is highly unlikely due to different cellular locations.

The final total protein yield of purified mFIZZ1 varies from 200 to 300 µg, which is relatively low due to the short translational life span of the wheat germ extract compared to cell-based expression [22]. For scaling-up the production using wheat germ extracts, an automatized system might be required [21]. Alternatively, co-expression with hQSOX1b [42] and/or hPDI in the *E. coli* might also be explored. This method is certainly cheaper and higher yields might be obtained, but here, the endotoxin levels are a drawback. When we tested the endotoxin level on purified expressed mFIZZ1′ in the limulus amebocyte lysate (LAL) assay, the same values as for PBS (negative control) were obtained (data not shown). So, the use of a bacteria-free expression system also overcomes the potential effects of LPS and other endotoxin contaminants, which is certainly an advantage for follow-up animal and/or cell culture testing.

We would like to emphasize that with the addition of quiescin sulfhydryl oxidase hQSOX1b we have introduced a new method for the folding of disulfide rich eukaryotic proteins. The sulfhydryl oxidase hQSOX1b does not require any additional partners to introduce disulfides into proteins. Although, the reaction with substrate proteins results in the formation of H_2_O_2_, and as a consequence oxidative stress, quiescin sulfhydryl oxidases are the most competent catalysts known for the *de novo* insertion of protein disulfide bonds in the endoplasmic reticulum [15], [43]. We used mFIZZ1 and mFIZZ1′ as model proteins that potentially had to form multiple disulfides for bioactivity. So far, no soluble protein expression method was established for this immunological important protein and no structural data are available. Nguygen *et al.* [24] followed another methodological approach for the expression of disulfide rich proteins in the cytoplasm of *E. coli* with the help of sulfhydryl oxidase/disulfide isomerase Erv1p. They showed how Erv1p is capable of introducing multiple disulfide bonds in a fragment of tissue plasminogen activator (vtPA) and the homodimeric resistin (mFIZZ3). We use the open cell free expression system. Our wheat germ cell free expression method is simple and effective for the *in vitro* production of soluble and active recombinant eukaryotic proteins that have to form multiple disulfides. Moreover, an open system makes it easier to screen for the correct folding partners as the addition of multiple oxidases and/or isomerases becomes in vision.

## Materials and Methods

### mFIZZ1 cloning and expression in *E. coli*

The mFIZZ1 gene without signal peptide (D24-S111, GenBank accession number AF205951) was cloned into the pET-14b vector (Novagen) with an N-terminal His-tag MGSSHHHHHHSSGLVPRGSHM-mFIZZ1. The coding sequence of mFIZZ1 was amplified by PCR and introduced in pET14b restricted with NdeI and BamHI. The construct was sequenced at the VIB Genetic Service Facility (GSF). For periplasmic expression mFIZZ1 was fused to the PeIB signal sequence. Plasmid DNA of mFIZZ1 was transformed into *E. coli* SHuffle™ T7 Express (BioLabs), Origami™ DE3 and BL21 DE3 (Novagen), grown in LB medium supplemented with 25 µg/ml ampicillin, induced at a cell density (OD_600 nm_) of 0.7 with 1 mM isopropyl-b-D-thiogalactopyranoside (IPTG), and cultured for 6 h at 37°C except for SHuffle™ T7 cells, the expression done at 30°C. Harvested cells were resuspended in 50 mM potassium phosphate, pH 7.5, 300 mM NaCl, 0.1 mg/ml lysozyme, 0.1 mg/ml AEBSF, 0.1 µg/ml leupeptine. Cells were broken by sonication at 4°C and centrifuged for 30 min at 15,000 rpm. For identification, protein fractions of total (5 µl), supernatant (7.5 µl) and pellet (7.5 µl) were analyzed on non-reducing 15% SDS-PAGE and immunoblot using anti-His monoclonal antibody (Sigma Aldrich, Belgium).

### mFIZZ1, mFIZZ1′, hQSOX1b and hPDI cloning into pEU vector

mFIZZ1 (D24–S111) and mFIZZ1′(M1-S111,GenBank accession number AF205951) were cloned into the pEU-vector (CellFree Sciences, Matsuyama, Japan) with an N-terminal His-tag MGHHHHHHLE-mFIZZ1. This plasmid vector is specially designed for the wheat-germ cell-free expression system [21] in combination with the SP6 RNA polymerase transcription system. The coding sequence of mFIZZ1′ was amplified by PCR and introduced using XhoI and SmaI restriction sites. mFIZZ1 was amplified and cloned in the XhoI-digested pEU vector using InFusion technology (Clontech**)**. The hQSOX1b (R32-I604, GenBank accession number NP_001004128.1) without signal peptide and hPDI (A18-L508, GenBank accession number NP_000909.2) without signal peptide genes were cloned with a GST-tag at the N-terminal position into the pEU-GST-MCS vector. The coding sequence of hQSOX1b and hPDI were amplified by PCR and introduced into the pEU-GST-MCS vector digested with BamHI and SmaI, or the XhoI and SmaI, respectively.

All constructs were sequenced at the VIB Genetic Service Facility (GSF).

### Small-scale transcription and translation reaction

Plasmid DNA of mFIZZ1, mFIZZ1′, hPDI and hQSOX1b (2 µg) was transcribed using SP6 RNA polymerase, 25 mM NTP mix, RNase inhibitor and 5× transcription buffer (Cell Free Sciences, Matsuyama, Japan) for 6 h at 37°C. The mRNA was cooled down to avoid degradation, and checked on 1% agarose gel. For translation, 10 µl of mRNA was mixed with the same amount of the wheat germ extract WEPRO 7240 (CellFree Sciences, Matsuyama, Japan) and 0.1 mg of creatine kinase to make the bottom layer, and incubated with 206 µl of 1× SUB-A MIX SGC (upper layer) at 15°C for 20 h without shaking in a 6-well plate (Greiner bio-one, Belgium) in a Thermomixer (Roche, Germany). The reaction mixture was centrifuged (15,000 rpm) for 30 min at 4°C. For identification, protein fractions, total (5 µl), soluble (7.5 µl) and pellet (7.5 µl) of the expressed proteins were visualized on immunoblot using as primary antibody anti-His or anti-GST antibody (EnoGene, Germany) and as secondary anti mouse polyclonal antiserum (Sigma Aldrich, Belgium). The same samples were ran on a non-reducing 15% SDS-PAGE followed by Coomassie Brilliant Blue staining.

### Co-expression of mFIZZ1 with hQSOX1b and/or hPDI

pEU GST-tag hQSOX1b, GST-tag hPDI and His-tag mFIZZ1 or mFIZZ1′ (2 µg each) were separately transcribed using SP6 RNA polymerase, 25 mM NTP mix, RNase inhibitor and 5× transcription buffer. The mRNA of respectively mFIZZ1, mFIZZ1′, hQSOX1b and hPDI were translated for 10 min using the wheat germ extract WEPRO 7240 at 26°C. mRNA of mFIZZ1 or mFIZZ1′ (10 µl each) was then mixed with the same amount of mRNA from hQSOX1b, hPDI, and hQSOX1b + hPDI and incubated with 206 µl of the SUB-A mixture for each reaction at 15°C for 20 h without shaking in a 96-well plate. After the incubation, the reaction mixture was centrifuged at 15,000 rpm for 30 min at 4°C. The protein concentration of the soluble and pellet fractions was determined using a Bradford assay [44]. A same amount (30 µg) of pellet and soluble proteins were ran on a non-reducing 15% SDS-PAGE and visualized by immunoblot using anti-His (Sigma Aldrich, Belgium) and anti-GST antibody (EnoGene, Germany). All bands from the immunoblots were scanned and the percentage was determined using Labimage programe (http://www.labimage.com). The experiments were repeated three times for reproducibility.

### Upscaling of the mFIZZ1 and mFIZZ1′ production with and without hQSOX1b

For up-scaling, plasmid DNA of His-tag mFIZZ1 and mFIZZ1′ (25 µg) and (25 µg) GST-tag hQSOX1b were transcribed separately using SP6 RNA polymerase, 25 mM NTP mix, RNase inhibitor and 5× transcription buffer for 6 h at 37°C. For translation, 250 µl of mRNA was mixed with the same amount of wheat germ extract (WEPRO 7240), and with 1 mg of creatine kinase to make the bottom layer. 5.5 ml of 1× SUB-A MIX included a mixture of amino acids were used to make the upper layer. Translation was performed at 15°C for 20 h without shaking in a 6-well plate using a Thermomixer. The expressed proteins were detected by immunoblotting using anti-His antibody (Sigma Aldrich, Belgium) and on CBB-stained 15% SDS-PAGE prior to purification.

For co-expression, mRNA from mFIZZ1 or mFIZZ1′ and hQSOX1b were translated for 10 min at 26°C using wheat germ extract WEPRO 7240H. After 10 min incubation, mRNA of mFIZZ1 or mFIZZ1′ were mixed with the same amount of mRNA from hQSOX1b and translated at 15°C for 20 h without shaking.

For purification, 2 batches (mFIZZ1 or mFIZZ1′) of 6 ml reaction (with and without hQSOX1b) were centrifuged at 15,000 rpm for 15 min at 4°C. The supernatant was separately loaded on 1 ml His-trap Ni-NTA resin equilibrated with 50 mM potassium phosphate buffer solution pH 7.5, 150 mM NaCl containing 10 mM imidazole. The column was washed with 5 column volumes and, the protein was eluted with a linear imidazole gradient from 50 to 500 mM in the same buffer solution. The purity of the elution peak fractions was evaluated on 15% SDS-PAGE under reducing and non- reducing conditions. Pure fractions were collected and dialyzed against PBS for 4 h at 4°C with two buffer changes. Protein concentrations were spectrophotometrically determined with a molar extinction of 18,740 M^−1^ cm^−1^ at 280 nm. Protein aliquots were stored at −20°C.

### Basic-native gel protocol

For the basic-native gel conditions, a method for acidic and neutral proteins was used (http://wolfson.huji.ac.il/purification/Protocols/PAGE_Basic.html). Briefly, the samples of mFIZZ1 (pI 4.81) and mFIZZ1′ (pI 5.18) expressed with and without hQSOX1b were mixed on ice with sample buffer solution containing 100 mM Tris/HCl, pH 6.8, bromophenol blue, and glycerol. For the reduced conditions, the samples were first incubated for 30 min with 20 mM DTT. Samples were loaded on a polyacrylamide native gel (5% stacking gel (pH 6.8) and 15% resolving gel (pH 8.9)). The running buffer solution contained 50 mM Tris/HCl, pH 8.9, and 380 mM glycine. As marker the PageRuler™ pre-stained Protein Ladder (Fermentas) is used, which contains SDS. After a 5 h run at 4°C, gels were CBB stained.

### Cross-linking conditions

Samples of mFIZZ1 (10 µM), mFIZZ1′ (5.3 µM), mFIZZ1 + hQSOX1b (20 µM), and mFIZZ1′ + hQSOX1b (9.2 µM) were were incubated with the cross-linker mixture of 1 mM EDC and 1 mM NHS (Pierce, Belgium) for 3 h at room temperature in PBS, pH 7.4 buffer solution. The reaction was stopped by addition of SDS-PAGE sample buffer. After boiling for 10 minutes, the mixture was evaluated on 15% SDS-PAGE under non-reducing conditions. As negative control, monomeric *Corynebacterium glutamicum* mycoredoxin-1 (Cg_Mrx1) [45], [46] was used. As positive control, dimeric *Escherichia coli* DsbG (Ec_DsbG) [8], [47] was used.

### Circular dichroism (CD) and mass spectrophotometry identification

CD-spectra were recorded at 25°C using a spectropolarimeter (Jasco, model 715, Tokyo, Japan). Protein was dissolved in water at 0.2 mg/ml. CD-spectra were recorded at a scan speed of 50 nm/min, 1 nm bandwidth and with a response time of 1 s. Spectra were recorded between in 0.01 cm path length quartz cuvette from 190 to 260 nm. The mFIZZ1 protein band was excised from the gel and the tryptic peptides were analyzed by tandem mass spectrometry (MS/MS) as described [48]. The percentage of α-helical and β-strand content was determined by sending the recorded spectra (190–260 nm) to the Dichroweb server (http://dichroweb.cryst.bbk.ac.uk) [29].We tested the different available algorithms. The CDSSTR algorithm [28] gave the lowest nmrsd values and the best fit to our experimental data.

### Disulfide bond formation assay

The number of free thiols in samples was determined using a Thiostar assay (Detect X^tm^, Luminos) [42], [49]. A standard curve of reduced L-glutathione (Sigma) ranging from 0 to 6 µM in a 96-well plate was prepared in water. mFIZZ1 and mFIZZ1′ samples expressed with and without hQSOX1b (5 µM) were 10 times diluted in water. After mixing with 15 µl of Thiostar reagent, the plate was incubated for 30 min in the dark, prior to measure at 510 nm with excitation at 390 nm in a fluorescent plate reader (Infinite M200, TECAN).

### Expression, and purification of DsbA, DsbC, hQSOX1b and hPDI

Expression and purification of DsbA and DsbC were performed as mentioned [32], hQSOX1b [43] and hPDI [37]. Purified DsbA, DsbC and hPDI were stored at −20°C. The purified hQSOX1b was stored at 4°C in the dark.

### Bioactivity assay of mFIZZ1

Single cell suspensions from C57BL/6 mouse spleens were cultured under Th2 permissive conditions with the addition of PBS (control), bacterial recombinant mFIZZ1/RELMα (Peprotech), or mFIZZ1′ expressed with or without hQSOX1b at concentrations indicated. Peprotech RELMα was generated in *E. coli* according to standard bacterial expression systems, and in the absence of any specific protocols to ensure disulfide bond formation (see www.peprotech.com for more information). Protein purity was confirmed by SDS-PAGE and HPLC analyses. Cells were cultured at the concentration of 2×10^5^ cells/well in 96-well round-bottom plates. Th2-permissive conditions were: αCD3/αCD28 (1 µg/mL each, eBioscience), rIL-4 (40 ng/mL; eBioscience), anti-IL-12 (10 µg/mL; clone C17.8) and anti-IFNγ (10 µg/mL; clone XMG 1.2). Four days later, supernatants were recovered for quantification of IL-5 and IL-13 by standard sandwich ELISA protocols (antibodies from eBioscience). Results are shown +/− S.D. and are representative of two or three independent experiments with quadruplicate wells per condition. Statistical significance was determined by using two-way anova analysis with treatment and experiment repeats as variables.

### RNase I activity assay

The RNA hydrolysis activity was performed as described [32]. RNA solution was mixed with the methylene blue buffer to obtain a final concentration of 450 µM RNA. After pre-incubation for 3 min at 25°C in the dark, *E. coli* RNase I was added. The absorbance change was followed in function of time at 659 nm, the wavelength with a maximum spectral difference between methylene blue intercalated RNA with and without RNase I.

### Oxidase, isomerase and chaperone assay

For the chaperone activity, unfolded RNase I was prepared by mixing guanidine hydrochloride (GuHCl), 50 mM Tris/HCl pH 8.0 and RNase I wild type to obtain the final concentration of 1.2 µM RNase I, 6.7 M GuHCl, 56 mM Tris/HCl pH 8.0. After 2 h incubation at 25°C, the mixture was dialyzed for 2 h against 0.6 M GuHCl, 50 mM Tris pH 8.7, 50 mM NaCl. Unfolded RNase I (500 nM) was pre-incubated with varying concentrations of (5 µM–50 nM) hQSOX1b, 5 µM DsbA or 5 µM DsbC in 50 mM Hepes/NaOH, pH 7.4, 150 mM NaCl for 3 min at 15°C before RNase activity was measured.

For the isomerase activity, *E. coli* scrambled RNase I (scRNase I) [32] was pre-incubated with 5 µM hQSOX1b, 5 µM DsbA, or 5 µM DsbC in 50 mM Hepes pH 7.4, 150 mM NaCl for 3 min at 15°C at a final concentration of 0.5 µM scRNase before measuring RNase activity.

For the oxidase assay, reduced and unfolded RNase I (ruRNase I) [32] was pre-incubated with the thiol/disulfide oxidoreductases in 50 mM Hepes/NaOH, pH 7.4, 150 mM NaCl for 3 min at 25°C at a final concentration of 1 µM ruRNase before measuring RNase activity. hPDI was reduced with 20 mM DTT for 1 h at 25°C. DTT was removed on a Superdex75 HR column (GE Healthcare Life Sciences) equilibrated in 50 mM Na phosphate, pH 7.5, 150 mM NaCl. Reduced and unfolded RNase I was pre-incubated with 5 µM hQSOX1b, 5 µM hPDI, 50 nM hQSOX1b +5 µM hPDI, 5 µM DsbA, 5 µM DsbC, 5 µM DsbA +5 µM DsbC, in 50 mM Hepes/NaOH, pH 7.4, 150 mM NaCl for 3 min at 25°C at a final concentration of 1 µM ruRNase I, before measuring RNase I activity.

## References

[1] Holcomb IN, Kabakoff RC, Chan B, Baker TW, Gurney A, et al (2000) FIZZ1, a novel cysteine-rich secreted protein associated with pulmonary inflammation, defines a new gene family. EMBO J 19: 4046–4055. 10921885 10.1093/emboj/19.15.4046 PMC306596

[2] Nair MG, Guild KJ, Artis D (2006) Novel effector molecules in type 2 inflammation: lessons drawn from helminth infection and allergy. J Immunol 177: 1393–1399. 16849442 10.4049/jimmunol.177.3.1393 PMC1780267

[3] Nair MG, Gallagher IJ, Taylor MD, Loke P, Coulson PS, et al (2005) Chitinase and Fizz family members are a generalized feature of nematode infection with selective upregulation of Ym1 and Fizz1 by antigen-presenting cells. Infect Immun 73: 385–394. 15618176 10.1128/IAI.73.1.385-394.2005 PMC538942

[4] Patel SD, Rajala MW, Rossetti L, Scherer PE, Shapiro L (2004) Disulfide-dependent multimeric assembly of resistin family hormones. Science 304: 1154–1158. 15155948 10.1126/science.1093466

[5] Sandler NG, Mentink-Kane MM, Cheever AW, Wynn TA (2003) Global gene expression profiles during acute pathogen-induced pulmonary inflammation reveal divergent roles for Th1 and Th2 responses in tissue repair. J Immunol 171: 3655–3667. 14500663 10.4049/jimmunol.171.7.3655

[6] Depuydt M, Messens J, Collet JF (2011) How proteins form disulfide bonds. Antioxid Redox Signal 15: 49–66. 20849374 10.1089/ars.2010.3575

[7] Wouters MA, Fan SW, Haworth NL (2010) Disulfides as redox switches: from molecular mechanisms to functional significance. Antioxid Redox Signal 12: 53–91. 19634988 10.1089/ars.2009.2510

[8] Messens J, Collet JF (2006) Pathways of disulfide bond formation in *Escherichia coli* . Int J Biochem Cell Biol 38: 1050–1062. 16446111 10.1016/j.biocel.2005.12.011

[9] Riemer J, Bulleid N, Herrmann JM (2009) Disulfide formation in the ER and mitochondria: two solutions to a common process. Science 324: 1284–1287. 19498160 10.1126/science.1170653

[10] Karala AR, Lappi AK, Ruddock LW (2010) Modulation of an active-site cysteine pKa allows PDI to act as a catalyst of both disulfide bond formation and isomerization. J Mol Biol 396: 883–892. 20026073 10.1016/j.jmb.2009.12.014

[11] Tu BP, Ho-Schleyer SC, Travers KJ, Weissman JS (2000) Biochemical basis of oxidative protein folding in the endoplasmic reticulum. Science 290: 1571–1574. 11090354 10.1126/science.290.5496.1571

[12] Sevier CS, Cuozzo JW, Vala A, Aslund F, Kaiser CA (2001) A flavoprotein oxidase defines a new endoplasmic reticulum pathway for biosynthetic disulphide bond formation. Nat Cell Biol 3: 874–882. 11584268 10.1038/ncb1001-874

[13] Thorpe C, Hoober KL, Raje S, Glynn NM, Burnside J, et al (2002) Sulfhydryl oxidases: emerging catalysts of protein disulfide bond formation in eukaryotes. Arch Biochem Biophys 405: 1–12. 12176051 10.1016/s0003-9861(02)00337-5

[14] Coppock DL, Kopman C, Scandalis S, Gilleran S (1993) Preferential gene expression in quiescent human lung fibroblasts. Cell Growth Differ 4: 483–493. 8396966

[15] Kodali VK, Thorpe C (2010) Oxidative protein folding and the Quiescin-sulfhydryl oxidase family of flavoproteins. Antioxid Redox Signal 13: 1217–1230. 20136510 10.1089/ars.2010.3098 PMC2959182

[16] Wittke I, Wiedemeyer R, Pillmann A, Savelyeva L, Westermann F, et al (2003) Neuroblastoma-derived sulfhydryl oxidase, a new member of the sulfhydryl oxidase/Quiescin6 family, regulates sensitization to interferon gamma-induced cell death in human neuroblastoma cells. Cancer Res 63: 7742–7752. 14633699

[17] Coppock DL, Cina-Poppe D, Gilleran S (1998) The quiescin Q6 gene (QSCN6) is a fusion of two ancient gene families: thioredoxin and ERV1. Genomics 54: 460–468. 9878249 10.1006/geno.1998.5605

[18] Powis G, Montfort WR (2001) Properties and biological activities of thioredoxins. Annu Rev Pharmacol Toxicol 41: 261–295. 11264458 10.1146/annurev.pharmtox.41.1.261

[19] Lisowsky T (1992) Dual function of a new nuclear gene for oxidative phosphorylation and vegetative growth in yeast. Mol Gen Genet 232: 58–64. 1552903 10.1007/BF00299137

[20] Alon A, Heckler EJ, Thorpe C, Fass D (2010) QSOX contains a pseudo-dimer of functional and degenerate sulfhydryl oxidase domains. FEBS Lett 584: 1521–1525. 20211621 10.1016/j.febslet.2010.03.001

[21] Takai K, Sawasaki T, Endo Y (2010) Practical cell-free protein synthesis system using purified wheat embryos. Nature protocols 5: 227–238. 20134421 10.1038/nprot.2009.207

[22] Endo Y, Sawasaki T (2006) Cell-free expression systems for eukaryotic protein production. Curr Opin Biotechnol 17: 373–380. 16828277 10.1016/j.copbio.2006.06.009

[23] Juan CC, Kan LS, Huang CC, Chen SS, Ho LT, et al (2003) Production and characterization of bioactive recombinant resistin in *Escherichia coli* . J Biotechnol 103: 113–117. 12814870 10.1016/s0168-1656(03)00099-3

[24] Nguyen VD, Hatahet F, Salo KE, Enlund E, Zhang C, et al (2011) Pre-expression of a sulfhydryl oxidase significantly increases the yields of eukaryotic disulfide bond containing proteins expressed in the cytoplasm of *E. coli* . Microb Cell Fact 10: 1. 21211066 10.1186/1475-2859-10-1 PMC3022669

[25] Updegrove TB, Correia JJ, Chen Y, Terry C, Wartell RM (2011) The stoichiometry of the Escherichia coli Hfq protein bound to RNA. RNA 17: 489–500. 21205841 10.1261/rna.2452111 PMC3039148

[26] Panchaud A, Hansson J, Affolter M, Bel Rhlid R, Piu S, et al (2008) ANIBAL, stable isotope-based quantitative proteomics by aniline and benzoic acid labeling of amino and carboxylic groups. Mol Cell Proteomics 7: 800–812. 18083701 10.1074/mcp.M700216-MCP200

[27] Banerjee RR, Lazar MA (2001) Dimerization of resistin and resistin-like molecules is determined by a single cysteine. J Biol Chem 276: 25970–25973. 11358969 10.1074/jbc.M103109200

[28] Sreerama N, Woody RW (2000) Estimation of protein secondary structure from circular dichroism spectra: comparison of CONTIN, SELCON, and CDSSTR methods with an expanded reference set. Anal Biochem 287: 252–260. 11112271 10.1006/abio.2000.4880

[29] Whitmore L, Wallace BA (2004) DICHROWEB, an online server for protein secondary structure analyses from circular dichroism spectroscopic data. Nucleic Acids Res 32: W668–673. 15215473 10.1093/nar/gkh371 PMC441509

[30] Nair MG, Du Y, Perrigoue JG, Zaph C, Taylor JJ, et al (2009) Alternatively activated macrophage-derived RELM-{alpha} is a negative regulator of type 2 inflammation in the lung. J Exp Med 206: 937–952. 19349464 10.1084/jem.20082048 PMC2715126

[31] Munitz A, Seidu L, Cole ET, Ahrens R, Hogan SP, et al (2009) Resistin-like molecule alpha decreases glucose tolerance during intestinal inflammation. J Immunol 182: 2357–2363. 19201890 10.4049/jimmunol.0803130 PMC2653277

[32] Messens J, Collet JF, Van Belle K, Brosens E, Loris R, et al (2007) The oxidase DsbA folds a protein with a nonconsecutive disulfide. J Biol Chem 282: 31302–31307. 17702751 10.1074/jbc.M705236200

[33] Depuydt M, Messens J, Collet JF (2011) How proteins form disulfide bonds. Antioxid Redox Signal 15: 49–66. 20849374 10.1089/ars.2010.3575

[34] Zapun A, Missiakas D, Raina S, Creighton TE (1995) Structural and functional characterization of DsbC, a protein involved in disulfide bond formation in *Escherichia coli* . Biochemistry 34: 5075–5089. 7536035 10.1021/bi00015a019

[35] Hatahet F, Ruddock LW (2009) Protein disulfide isomerase: a critical evaluation of its function in disulfide bond formation. Antioxid Redox Signal 11: 2807–2850. 19476414 10.1089/ars.2009.2466

[36] Grynberg A, Nicolas J, Drapron R (1977) Presence of a protein disulfide isomerase (EC 5.3.4.1) in wheat germ. C R Acad Sci Hebd Seances Acad Sci D 284: 235–238. 404057

[37] Rancy PC, Thorpe C (2008) Oxidative protein folding in vitro: a study of the cooperation between quiescin-sulfhydryl oxidase and protein disulfide isomerase. Biochemistry 47: 12047–12056. 18937500 10.1021/bi801604x PMC2892342

[38] Paetzel M, Karla A, Strynadka NC, Dalbey RE (2002) Signal peptidases. Chem Rev 102: 4549–4580. 12475201 10.1021/cr010166y

[39] Wilson R, Lees JF, Bulleid NJ (1998) Protein disulfide isomerase acts as a molecular chaperone during the assembly of procollagen. J Biol Chem 273: 9637–9643. 9545296 10.1074/jbc.273.16.9637

[40] Cai H, Wang CC, Tsou CL (1994) Chaperone-like activity of protein disulfide isomerase in the refolding of a protein with no disulfide bonds. J Biol Chem 269: 24550–24552. 7929125

[41] Wang C, Yu J, Huo L, Wang L, Feng W, et al (2012) Human protein-disulfide isomerase is a redox-regulated chaperone activated by oxidation of domain a'. J Biol Chem 287: 1139–1149. 22090031 10.1074/jbc.M111.303149 PMC3256865

[42] Abskharon RN, Ramboarina S, El Hassan H, Gad W, Apostol MI, et al (2012) A novel expression system for production of soluble prion proteins in *E. coli* . Microb Cell Fact 11: 6. 22233534 10.1186/1475-2859-11-6 PMC3283519

[43] Heckler EJ, Alon A, Fass D, Thorpe C (2008) Human quiescin-sulfhydryl oxidase, QSOX1: probing internal redox steps by mutagenesis. Biochemistry 47: 4955–4963. 18393449 10.1021/bi702522q PMC3542536

[44] Bradford MM (1976) A rapid and sensitive method for the quantitation of microgram quantities of protein utilizing the principle of protein-dye binding. Anal Biochem 72: 248–254. 942051 10.1016/0003-2697(76)90527-3

[45] Van Laer K, Buts L, Foloppe N, Vertommen D, Van Belle K, et al (2012) Mycoredoxin-1 is one of the missing links in the oxidative stress defense mechanism of Mycobacteria. Mol Microbiol 86: 787–804. 22970802 10.1111/mmi.12030

[46] Ordóñez E, Van Belle K, Roos G, De Galan S, Letek M, et al (2009) Arsenate reductase, mycothiol, and mycoredoxin concert thiol/disulfide exchange. J Biol Chem 284: 15107–15116. 19286650 10.1074/jbc.M900877200 PMC2685692

[47] Depuydt M, Leonard SE, Vertommen D, Denoncin K, Morsomme P, et al (2009) A periplasmic reducing system protects single cysteine residues from oxidation. Science 326: 1109–1111. 19965429 10.1126/science.1179557

[48] Plesa M, Hernalsteens JP, Vandenbussche G, Ruysschaert JM, Cornelis P (2006) The SlyB outer membrane lipoprotein of Burkholderia multivorans contributes to membrane integrity. Res Microbiol 157: 582–592. 16500084 10.1016/j.resmic.2005.11.015

[49] Park S, Lippard SJ (2011) Redox state-dependent interaction of HMGB1 and cisplatin-modified DNA. Biochemistry 50: 2567–2574. 21355578 10.1021/bi2000214 PMC3068233

[50] Thompson JD, Higgins DG, Gibson TJ (1994) CLUSTAL W: improving the sensitivity of progressive multiple sequence alignment through sequence weighting, position-specific gap penalties and weight matrix choice. Nucleic Acids Res 22: 4673–4680. 7984417 10.1093/nar/22.22.4673 PMC308517

